# Liver-specific FGFR4 knockdown in mice on an HFD increases bile acid synthesis and improves hepatic steatosis

**DOI:** 10.1016/j.jlr.2022.100324

**Published:** 2022-12-29

**Authors:** Francois Moreau, Bruna Brasil Brunao, Xiang-Yu Liu, Frederic Tremblay, Kevin Fitzgerald, Julian Avila-Pacheco, Clary Clish, Ronald C. Kahn, Samir Softic

**Affiliations:** 1 Section of Integrative Physiology and Metabolism, Joslin Diabetes Center, Harvard Medical School, Boston, MA, USA; 2 Alnylam Pharmaceuticals, Cambridge, MA, USA; 3 Metabolomics Platform of the Broad Institute of MIT and Harvard, Cambridge, MA, USA; 4 Division of Gastroenterology, Hepatology and Nutrition, Department of Pediatrics, and Department of Pharmacology and Nutritional Sciences, University of Kentucky College of Medicine, University of Kentucky, Lexington, KY, USA

**Keywords:** fibroblast growth factor receptor 4, farnesoid X receptor, cholesterol, bile acids, liver steatosis, NAFLD, Insulin Resistance, Obesity, Liver, IRS-1, FGFR, fibroblast growth factor receptor, FXR, farnesoid X receptor, GalNAc, N-acetylgalactosamine, HFD, high-fat diet, HOMA-IR, Homeostatic Model Assessment of Insulin Resistance, IR, insulin receptor, KD, knockdown, NAFLD, nonalcoholic fatty liver disease, qPCR, quantitative PCR, RNA-seq, RNA-sequence, SHP, small heterodimer partner

## Abstract

Nonalcoholic fatty liver disease (NAFLD) is the most common chronic liver disease with increased risk in patients with metabolic syndrome. There are no FDA-approved treatments, but FXR agonists have shown promising results in clinical studies for NAFLD management. In addition to FXR, fibroblast growth factor receptor FGFR4 is a key mediator of hepatic bile acid synthesis. Using N-acetylgalactosamine–conjugated siRNA, we knocked down FGFR4 specifically in the liver of mice on chow or high-fat diet and in mouse primary hepatocytes to determine the role of FGFR4 in metabolic processes and hepatic steatosis. Liver-specific FGFR4 silencing increased bile acid production and lowered serum cholesterol. Additionally, we found that high-fat diet–induced liver steatosis and insulin resistance improved following FGFR4 knockdown. These improvements were associated with activation of the FXR-FGF15 axis in intestinal cells, but not in hepatocytes. We conclude that targeting FGFR4 in the liver to activate the intestinal FXR-FGF15 axis may be a promising strategy for the treatment of NAFLD and metabolic dysfunction.

Nonalcoholic fatty liver disease (NAFLD) is characterized by excessive lipid accumulation in the liver (1). It is the most common chronic liver diseases (2) and the second most common cause of liver transplant in the United States (3). Severe obesity, type 2 diabetes, and hypercholesterolemia are important risk-factors that increase liver-related morbidity (4). However, cardiovascular complications are the most common cause of increased mortality in patients with NAFLD.

In spite of enormous disease burden, there are no approved pharmacotherapies for the treatment of NAFLD. The FXR agonist, obeticholic acid, is one of the leading candidates that showed promising phase 3 results in patients with advanced NAFLD (5). FXR is a nuclear receptor that suppresses cholesterol 7 alpha-hydroxylase (CYP7A1), the rate-limiting enzyme in bile acid synthesis from cholesterol (6). This is mediated via FXR induction of small heterodimer partner (SHP), which then inhibits the transcription of *Cyp7a1* gene. As expected, obeticholic acid decreases bile acid synthesis but increases serum cholesterol (7). Another pathway that controls CYP7A1 activity is via fibroblast growth factor and their respective receptors (fibroblast growth factor receptor, FGFR) (8).

There are four genes that code for FGFR in humans, *FGFR 1*, *2*, *3*, and *4* (9, 10). Due to alternative RNA splicing, several FGFR isoforms were described (11, 12, 13). All FGFR share a common structure that consists of three extracellular immunoglobulin domains, a transmembrane helix domain, and an intracellular tyrosine kinase domain (14). In mice, both *Fgfr1* and *2* are expressed in most of the tissues, while *Fgfr3* expression is isoform dependent (15) and *Fgfr4* is expressed in the kidney, liver, adrenal, and lung.

In the liver, FGFR4 and FXR are master regulators of the bile acid synthesis (16, 17). Once synthesized in the liver, bile acids are secreted into the intestinal lumen to facilitate the absorption of dietary fats (18). Bile acids are then reabsorbed in the ileum and activate intestinal nuclear receptors FXR and TGR5 (19). The activation of FXR leads to the intestinal secretion of FGF15 in mice or FGF19 in humans. FGF15 circulates to the liver to bind and activate FGFR4 that then inhibits *Cyp7a1* expression and reduces further bile acids synthesis. Whole-body deletion of FGFR4 improves insulin sensitivity, glucose metabolism, and liver steatosis (20, 21). However, FGFR4 activation or overexpression specifically in the liver has been reported to have no effect on glucose metabolism and insulin sensitivity (21, 22).

In this study, we have investigated the liver-specific role of FGFR4 on hepatic steatosis, lipid, and glucose metabolism. We used an siRNA conjugated with a trivalent *N*-acetylgalactosamine (GalNAc) to knockdown (KD) FGFR4 in the liver (23, 24). GalNAc binds to the asialoglycoprotein receptor, highly expressed on hepatocyte, leading to a hepatocyte-specific delivery of the siRNA and thus to a liver-specific KD (25). We show that a liver FGFR4 silencing increases *Cyp7a1* expression and elevates hepatic bile acids. This results in decreased serum cholesterol, improved hepatic steatosis, and enhanced insulin sensitivity. Mechanistically increased bile acids stimulate intestinal FXR-FGF15 axis, while the liver FXR activity remains unchanged. FGFR4 KD in the liver improves steatosis and metabolic disfunction and thus may be a promising therapeutic target for the treatment of NAFLD.

## Materials and Methods

### Animals and diets

Mice were housed 4 per cage, at 20–22 degree Celsius on a 12 h light / 12 h dark cycle. Six-week-old C57Bl/6J male mice were purchased from The Jackson Laboratory and placed either on a chow diet (23% protein, 21.6% fat, and 55.4% carbohydrates, Mouse Diet 9F, PharmaServ) or an high-fat diet (HFD, 20% protein, 60% fat, and 20% carbohydrates, Research diets, D12492) for 12 weeks. Mice had access to water and food ad libitum. Liver-specific FGFR4 KD was obtained using an siRNA conjugated with a GalNAc (23, 24) (Alnylam Pharmaceuticals). Mice were injected subcutaneously, every two weeks, with 3 mg/kg of siRNA or a sterile 0.9% sodium chloride solution as a control for 12 weeks. Body weight was measured weekly. All animal studies were approved by the IACUC of the Joslin Diabetes Center and were in accordance with NIH guidelines.

### Glucose and insulin tolerance test

Glucose tolerance was assessed by injecting 1 mg/kg of dextrose after a 2 h fast as previously described (26). Blood glucose was measured with a glucometer (Infinity, US Diagnostics), from the tail vein at time 0, 15, 30, 60, and 120 min following IP glucose injection. Insulin sensitivity was assessed by injecting (i.p.) 1 IU/kg of insulin in random fed mice. Blood glucose was measured at 0, 15, 30, 60, and 90 min after insulin injection.

### Liver histology and triglyceride quantification

All the tissues were collected and snap frozen in liquid nitrogen or fixed into formalin. Tissues dedicated for histology were stained with hematoxylin and eosin. Liver histology was graded by a board-certified veterinary pathologist blinded to the experimental conditions. Triglycerides from liver samples were measured in accordance to methods previously published (27). Briefly, 100 mg of liver tissue was collected and homogenized in 1 ml of a 2:1 v/v mix of chloroform-methanol. After a centrifugation at 15,000 *g* for 15 min at 4°, the supernatant (10 μl) was transferred in a glass tube and evaporated for 1–2 h at room temperature. Evaporated lipid was resuspended in 200 μl of Triglycerides Reagent (Pointe Scientific, catalog T7532) and incubated at room temperature for 15 min. The absorbance was measured at 500 nm wavelength. The concentration was calculated using the standard curve generated from the serial dilution of a 200 mg/dl triglyceride standard.

### Quantification of plasma cholesterol, total bile acids, and FGF15 levels

Plasma samples were collected at the time of the sacrifice by cardiac puncture in an EDTA-treated tube. Cholesterol was measured using a total cholesterol colorimetric assay kit (Cell biolabs, #STA-384) following the protocol supplied by the manufacturer. Briefly, plasma samples were diluted (1:100) in the sample buffer supplied. Absorbance was read at 450-nm and concentration was calculated using a 10 mM cholesterol standard. Total bile acids were quantified using a total bile acids colorimetric assay kit (Cell biolabs, #STA-631) and following the protocol supplied by the manufacturer. Briefly, undiluted plasma samples were used for this assay. Absorbance was read at 405-nm and 630-nm, and the concentration of total bile acids was calculated by subtracting the absorbance at 630-nm from the absorbance read at 405-nm. Total bile acids concentration was calculated using a 250 μM glycochenodeoxycholic acid standard. Circulating Fgf15 levels were assessed using a mouse Fgf15 Elisa kit (LifespanBioscience, #LS-F11446) by following the protocol supplied by the manufacturer. Briefly, each sample was diluted (1:2) with the sample buffer supplied. Absorbance was read at 450-nm and concentration was calculated using a 5000pg/ml FGF15 standard.

### Bile acids analysis

Bile acids were analyzed using a Nexera X2 U-HPLC system (Shimadzu Scientific Instruments; Marlborough, MA) coupled to a Q Exactive orbitrap mass spectrometer (Thermo Fisher Scientific; Waltham, MA). Plasma samples (30 μl) were extracted using 90 μl of methanol containing PGE2-d4 as an internal standard (Cayman Chemical Co.; Ann Arbor, MI) and centrifuged (10 min, 9,000 *g*, 4°C). The supernatants (10 μl) were injected onto a 150 × 2.1 mm ACQUITY BEH C18 column (Waters; Milford, MA). The column was eluted isocratically at a flow rate of 450 μl/min with 20% mobile phase A (0.01% formic acid in water) for 3 min followed by a linear gradient to 100% mobile phase B (0.01% acetic acid in acetonitrile) over 12 min. MS analyses were carried out using electrospray ionization in the negative ion mode using full scan analysis over m/z 70–850 at 70,000 resolution and 3 Hz data acquisition rate. Additional MS settings were ion spray voltage, −3.5 kV; capillary temperature, 320°C; probe heater temperature, 300°C; sheath gas, 45; auxiliary gas, 10; and S-lens RF level 60.

### mRNA, qPCR, and RNA-seq analysis

mRNA was extracted from liver and ileum biopsies in a mix of Trizol and chloroform and then precipitated with 70% of ethanol. mRNA purification was performed using RNeasy Mini Kit columns (QIAGEN, catalog 74,106). cDNA was produced by retrotranscription of mRNA. The quantitative PCR (qPCR) was performed on CFX384 Touch Real-Time PCR Detection System and the data were analyzed on CFX manager software (3.1, Bio-Rad). Primers sequences are listed in the supplemental Table S1. The HTG EdgeSeq mRNA sequence analysis was performed by BioPolymers Facility at Harvard Medical School. Reads were aligned to the mouse transcriptome with Kallisto, and the transcript counts were converted to gene counts with tximport. Genes with less than one count per million in more than 4 samples were excluded. Data were then normalized by trimmed mean of M-values. Normalization factors were between 0.83 and 1.2. To use linear models, data were converted to logCPM in Voom using the formula $CPM=10^{6\times count\;of\;a\;gene}/\left(total\;counts\;of\;the\;sample\times normalization\;factor\;of\;the\;sample\right)$. Data were analyzed on limma R package using the Fry function of the Rotation Gene set test (Roast) method. A moderated *t* test was performed to identify genes differentially expressed between FGFR4 and CTRL on different diets (*P*-value < 0.05 and FDR < 0.25).

### Mouse primary hepatocytes isolation

Mouse primary hepatocytes were isolated from C57Bl6J mice as described in (28). Briefly, mice were anesthetized with avertin before laparotomy. A canula was inserted into the inferior vena cava and a krebs solution containing 0.5 mM of EDTA and prewarmed at 37°C was perfused at a rate of 5 ml/min for 4 min. The portal vein was ligated to allow blood drainage. A digestion solution was prepared by adding 1.2 mg/ml of collagenase type 1 into DMEM high glucose. This solution was perfused at a rate of 5 ml/min for 8 min. The liver was next removed and transferred in a Petri dish containing 10 ml of the digestion solution and shaken for 3 min to release the cells. Cells freshly isolated were purified after a series of filtrations (100 μm) and centrifugations (50 *g*, 1min30, and 4C).

### FGFR4 silencing in mouse primary hepatocytes

Cells were seeded in a 12-well plate at a concentration of 200,000 cells/well in DMEM supplemented with of 1% antibiotics and 5% fetal bovine serum. The transfection of FGFR4 siRNA was performed using the lipofectamine RNAiMAX kit following the protocol supplied by the manufacturer. Briefly, the day after primary hepatocyte isolation, the complete media was removed and replaced by a media supplemented with 10% of fetal bovine serum. Cells were treated with siRNA (1000 ng/ml). On day 2 after hepatocyte isolation, half of the wells were treated for 24 h with free fatty acid mixture of oleic acids and palmitic acids (2:1) or only palmitic acid at a concentration of 500 μM. Every measure performed on these cells were done on day 3.

### Western blot

Proteins were extracted from primary hepatocyte and liver biopsies using a RIPA buffer (MilliporeSigma) supplemented with 0.1% SDS and a cocktail of protein phosphatase (#B15001-A and B15001-B, Bimake) and protease inhibitors (#B14001, Bimake) at a concentration of 1x. Protein concentrations were measured using a BCA protein assay kit (Pierce). Around 10 μg of protein was loaded in a 4%–12% Bis-Tris gel (Invitrogen) and then transferred to a PVDF membrane. The membrane was blocked at room temperature for 1 h in a blocking solution (Thermo Fisher Scientific) followed by an overnight incubation at 4°C with the primary antibodies. Membranes were washed in a TBS-T buffer followed by a 4 h incubation at room temperature with HRP-conjugated secondary antibodies. The following antibodies were used in this study: IRS-1 (catalog 2381) from BD Bioscience, β-actin (catalog 47778) from Santa Cruz, FGFR4 (catalog 8562), insulin receptor (IR, catalog 3025), p-IR^Y1150/1151^/IGF1R^Y1135/1136^ (catalog 3024), AKT (catalog 4685), p-AKT^Ser473^ (catalog 4060), ERK1/2 (catalog 9102), p-ERK1/2^T202/Y204^ (catalog 4370) and GAPDH (catalog 5174) from cell signaling, goat anti-rabbit HRP conjugated (catalog 1706515) from Bio-Rad, and sheep anti-mouse HRP conjugated (catalog NA931) from Sigma-Millipore.

### Statistics

All data are presented as mean ± SEM. Comparison between only two groups were made using a nonparametric test (Mann-whitney), while comparison between more than 2 groups were done by a two-way ANOVA followed by a Tukey’s multiple comparison test.

## Results

### Liver FGFR4 KD reduces HFD-induced liver steatosis

**Fig. 1:**
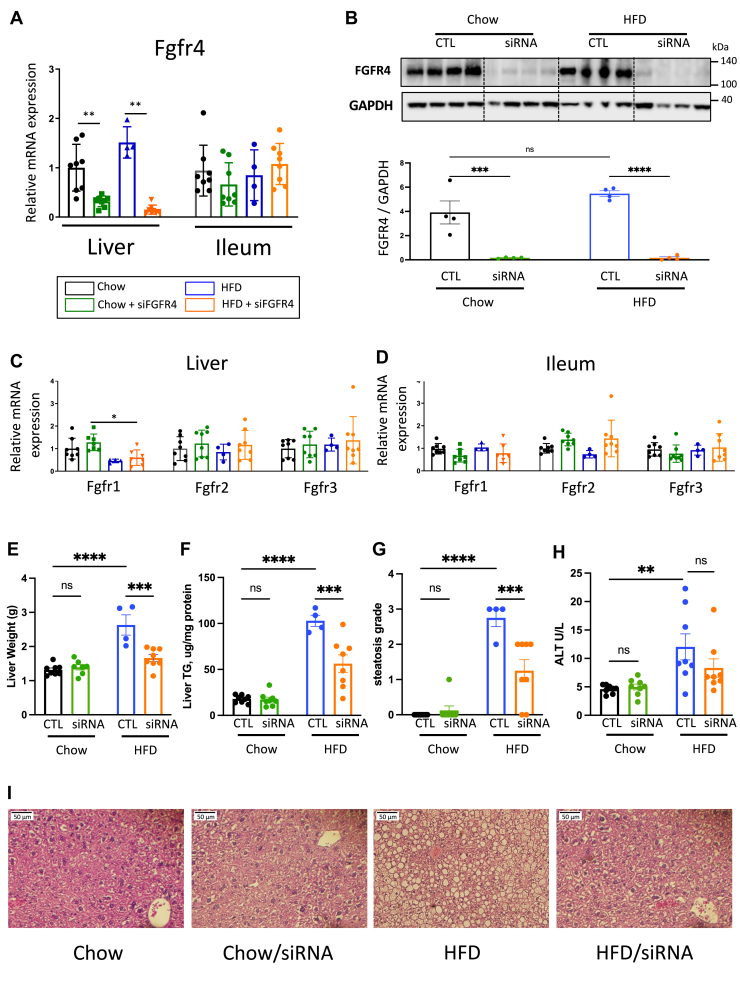
Liver FGFR4 KD improves liver steatosis. A: mRNA expression of *Fgfr4* in liver and ileum biopsies. Data were normalized by the gene expression in the chow diet group. B: Immunoblotting of FGFR4 from liver biopsies from mice receiving either a saline solution or 3 mg/kg of siRNA targeting FGFR4 and its quantitative analysis. Measure of *Fgfr1*, *2,* and *3* gene expressions from liver (C) and ileum (D) biopsies. Data were normalized by the gene expression in the chow diet group. Liver weight (E) and hepatic triglyceride (TG) content (F) after 12 weeks of diet. G: Liver steatosis score (0 = healthy, 1 = mild, 2 = moderate, and 3 = severe) calculated from hematoxylin and eosin (H&E)-stained liver (I). H: Serum levels of alanine transaminase (ALT). Data are expressed as mean ± SEM (n = 4–8, ns = non-significant, ∗ *P* < 0.05; ∗∗ *P*<0.01; ∗∗∗ *P* < 0.001; ∗∗∗∗*P* < 0.0001, two-way ANOVA followed by a Tukey’s multiple comparisons test). FGFR, fibroblast growth factor receptor; KD, knockdown.

Previous studies have shown that whole-body deletion of FGFR4 improves glucose metabolism, insulin sensitivity, and lipid accumulation in the liver of diet-induced obese mice (20, 21). Here, we sought to investigate the contribution of liver FGFR4 on hepatic steatosis in diet-induced obese mice. Six-week-old, male, C57Bl/6J mice were subjected to 12 weeks of chow or HFD feeding. FGFR4 siRNA was injected every 2 weeks. As expected, treatment with FGFR4 siRNA reduced *Fgfr4* mRNA expression by 3.2- and 10.8-fold in the livers of mice on chow or HFD, respectively (Fig. 1A). *Fgfr4* expression was unaffected in the ileum. In agreement with mRNA, the protein level of FGFR4 in the liver was almost completely abrogated in groups that received FGFR4 siRNA (Fig. 1B). To confirm tissue and target-specificity of the siRNA, we assessed the expression of *Fgfr4* and other members of the *Fgfr* family in liver and ileum of mice on chow and HFD. No changes were observed in the expression of *Fgfr 1, 2,* or *3* in the liver or in the intestine following FGFR4 KD (Fig. 1C). However, HFD decreased *Fgfr1* mRNA expression in the liver. In addition, FGFR4 cofactor *βKlotho* expression was unchanged following FGFR4 KD (supplemental Fig. S1A). Body weight was not affected by hepatic FGFR4 KD in Chow-fed mice and only modestly reduced in HFD-fed mice (supplemental Fig. S1B). In line with the increased body weight on HFD, calorie intake was higher in mice on HFD (supplemental Fig. S1C). FGFR4 KD slightly increased calorie intake in chow-fed mice, while it had no major effect on caloric intake in mice on HFD (supplemental Fig. S1C). On the other hand, liver weight was increased in HFD-fed mice and significantly decreased following FGFR4 KD (2.63 ± 0.29 g vs. 1.66 ± 0.10 g) (Fig. 1E). However, in mice on chow diet, liver weight was not affected by FGFR4 silencing (1.31 ± 0.05 g vs. 1.39 ± 0.07 g). The weight of subcutaneous, perigonadal, and brown adipose tissue was increased by an HFD, but it was not affected following FGFR4 KD (supplemental Fig. S1D–F). In addition, gastrocnemius, quadriceps, and tibialis anterior muscle weights were not significantly changed by either the diet or FGFR4 KD (supplemental Fig. S1G–I). In line with liver weight, hepatic TG content in mice on chow diet was similar with or without FGFR4 KD (17.88 ± 1.47 vs. 17.24 ± 2.61 μg/mg of liver) (Fig. 1F). Liver histology confirmed no steatosis in both cohorts of chow-fed mice (Fig. 1I). As expected, HFD significantly increased TG content in the liver by 5.7-fold (*P* = 0.004), which was reduced (*P* = 0.016) by hepatic FGFR4 KD. Despite the changes of hepatic TG content, serum TG levels were not affected by FGFR4 KD (supplemental Fig. S1J). Steatosis score, as graded by blinded pathologist, showed severe steatosis in HFD-fed mice, which was improved following FGFR4 KD (Fig. 1G). In line with liver steatosis, serum alanine transaminase was significantly increased by HFD and was partially restored by FGFR4 silencing (Fig. 1H).

### Liver FGFR4 KD improves insulin sensitivity

**Fig. 2:**
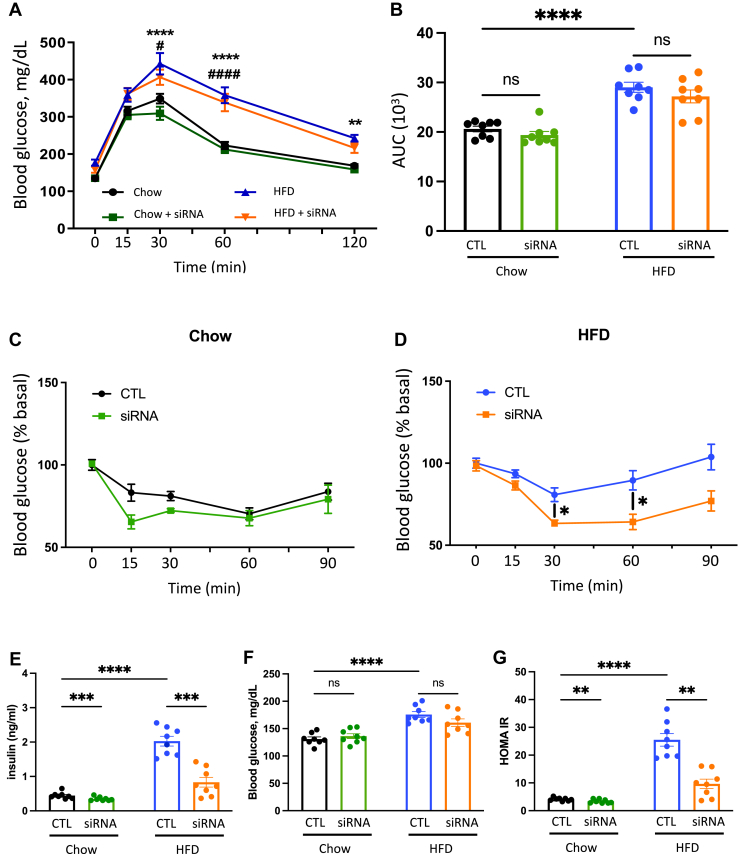
Liver FGFR4 KD improves insulin sensitivity in HFD-fed mice. A: Oral Glucose tolerance test (OGTT) performed after 7 weeks of diet. B: Area under the curve of the OGTT. Insulin tolerance test (ITT) performed after 10 weeks of diet in mice on chow (C) and HFD (D). G: Homeostatic Model Assessment of Insulin Resistance (HOMA-IR) measured after 11 weeks of diet and calculated from insulin levels (E) and blood glucose (F) after an overnight fasting (O/N). Data are expressed as mean ± SEM (n = 8, ns = non-significant, ∗ *P* < 0.05; ∗∗ *P* < 0.01; ∗∗∗ *P* < 0.001; ∗∗∗∗*P* < 0.0001, two-way ANOVA followed by a Tukey’s multiple comparisons test). FGFR, fibroblast growth factor receptor; HFD, high-fat diet; KD, knockdown.

Next, we investigated the impact of hepatic FGFR4 KD on insulin sensitivity and glucose tolerance. Oral glucose tolerance was not affected in chow-fed mice treated with or without FGFR4 siRNA (Fig. 2A, B). Glucose tolerance was impaired in mice on an HFD, but it was not improved with FGFR4 KD. Following exogenous insulin administration, blood glucose decreased in chow-fed control mice documenting normal insulin sensitivity (Fig. 2C). There was no effect on insulin sensitivity in FGFR4 siRNA-treated mice on a chow diet. While HFD impaired insulin sensitivity, FGFR4 siRNA treatment significantly improved insulin sensitivity (Fig. 2D). Insulin level was not affected by FGFR4 KD in chow-fed mice. As anticipated, fasted insulin levels were elevated in HFD-fed mice (2.02 ± 0.4 ng/ml) compared to the chow-fed controls (0.44 ± 0.1 ng/ml) (Fig. 2E). Consistent with the improved insulin sensitivity, insulin levels significantly decreased following FGFR4 KD in mice on an HFD (0.8 ± 0.4 ng/ml). In agreement with glucose tolerance, fasted blood glucose was not affected by FGFR4 KD in chow- or HFD-fed mice, but glucose was elevated in mice on an HFD (Fig. 2F). Insulin resistance, assessed by Homeostatic Model Assessment of Insulin Resistance (HOMA-IR), was significantly increased in HFD-fed mice and restored by KD of FGFR4 to the level observed in chow-fed mice (Fig. 2G). Taken together, KD of FGFR4 on chow diet had no effect on glucose tolerance and insulin sensitivity, while FGFR4 KD on an HFD resulted in improved insulin tolerance, lower fasted insulin, and improved HOMA-IR.

### Liver FGFR4 KD increases hepatic bile acids

**Fig. 3:**
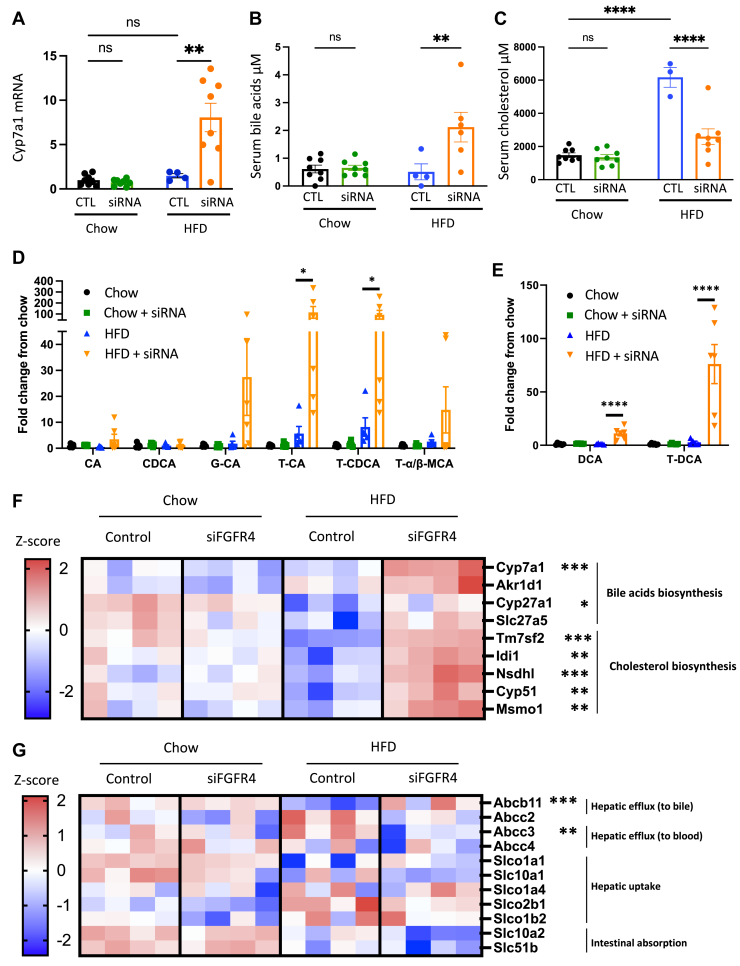
FGFR4 silencing in liver promotes bile acids synthesis. A: Hepatic gene expression of Cyp7A1. Measure of serum bile acids (B) and serum cholesterol (C). Measure of primary (D) and secondary (E) bile acids species in the cardiac blood. Data are expressed as mean ± SEM (n = 4–8, ns = non-significant, ∗ *P* < 0.05; ∗∗ *P* < 0.01; ∗∗∗∗*P* < 0.0001, two-way ANOVA followed by a Tukey’s multiple comparisons test). Data were normalized by the control condition in chow diet mice for (A, D, and E). F: Heatmap of cholesterol and bile acids biosynthesis genes expression. G: Heatmaps of genes involved in bile acids transport. Each column represents a mouse (n = 4, ∗ *P* < 0.05; ∗∗ *P*<0.01; ∗∗∗ *P*<0.001; moderated *t* test). FGFR, fibroblast growth factor receptor.

To investigate the underlying mechanism mediating improved hepatic steatosis and insulin sensitivity, we assessed the expression of downstream targets of FGFR4. *Cyp7a1* expression was not altered by FGFR4 KD in mice on a chow diet or by an HFD feeding. However, *Cyp7a1* expression increased 6.1-fold in HFD-fed mice treated with FGFR4 siRNA (Fig. 3A). We also assessed *Cyp7a1* expression after a short 2-weeks FGFR4 siRNA treatment in mice on an HFD. Despite a robust 73% decrease of *Fgfr4* expression, *Cyp7a1*, *Cyp7b1*, and *Fxr* mRNA was not changed by FGFR4 KD (supplemental Fig. S2A). These data suggest that obesity, rather than HFD, may be required to induce an increase in *Cyp7a1* expression following FGFR4 silencing. In line with *Cyp7a1* expression, hepatic CYP7A1 protein abundance tended to be increased by FGFR4 KD in HFD-fed mice (two-way ANOVA *P* = 0.3, Mann-Whitney *P* = 0.03) (supplemental Fig. S2B, C). In agreement with *Cyp7a1* mRNA, serum bile acids were unchanged following FGFR4 KD in mice on chow diet (Fig. 3B). On the other hand, FGFR4 KD in HFD-fed mice increased serum bile acids by 3.1-fold (Fig. 3B). Since liver cholesterol is a precursor for bile acids synthesis, we assessed the impact of FGFR4 KD on cholesterol levels. There was no difference in cholesterol levels with FGFR4 KD on chow diet. Mice on HFD exhibit a robust increase in serum cholesterol, and this was dramatically reversed by FGFR4 KD (Fig. 3C). A decrease in cholesterol is likely secondary to elevated Cyp7a1 and increased bile acids synthesis. To investigate this, we performed an untargeted LC-MS and observed that serum bile acids were similar in lean chow-fed FGFR4 KD mice compared to the control. However, suppression of FGFR4 in HFD-fed mice led to a significant increase in serum primary and secondary bile acids (Fig. 3D, E). Moreover, primary bile acids in the liver were not increased by FGFR4 silencing in chow-fed mice but were significantly increased in HFD-fed mice treated with FGFR4 siRNA (supplemental Fig. S2D, E). Together, these data suggest that downregulation of FGFR4 in the liver of obese mice facilitates the conversion of cholesterol to bile acids and this is largely due to the upregulation of Cyp7a1 leading to increased bile acid synthesis.

Next, we performed RNA sequence (RNA-seq) to comprehensively assess downstream pathways that mediate this process. Consistent with qPCR results, RNA-seq confirmed increased *Cyp7a1* mRNA, in HFD-fed mice treated with FGFR4 siRNA (Fig. 3F). Several additional genes involved in bile acids synthesis, such as *Akr1d1* and *Slc27a5*, tend to be increased, whereas Cyp27a1 was significantly increased in these mice (Fig. 3F). Moreover, *Cyp7b1* and *Cyp2c70* (supplemental Fig. S3B, C) and the two regulators of *Cyp7a1* gene expression, *Lrh1* and *Hnf4-α*, were unchanged by either the HFD or by silencing of FGFR4 (supplemental Fig. S3D, E). The genes mediating cholesterol de novo synthesis were not affected in mice on chow diet, but were reduced in mice on an HFD, in agreement with increased dietary cholesterol found in this lard-containing HFD (Fig. 3F). In contrast, the genes mediating cholesterol synthesis, such as *Tm7sf2* and *Nsdhl*, were strongly upregulated in HFD-fed mice following FGFR4 KD, in line with increased hepatic bile acid production (Figs. 3F and S4D, E). On the other hand, expression of the genes involved in bile acid secretion from liver to bile showed a profound increase in the expression of *Abcb11,* whereas expression of *Abcc2* tended to be decreased (*P*-value = 0.04, FDR = 0.47) (Figs. 3G and S4A, B). Moreover, expression of *Abcc3* involved in bile acid efflux from the liver to the blood was significantly downregulated in these mice (Figs. 3G and S4C). *Slc10a2* (*P*-value = 0.004, FDR = 0.57) and *Slc51b* (*P*-value = 0.01, FDR = 0.84)*,* involved in intestinal absorption, tended to be decreased (Figs. 3G and S4F). Genes regulating hepatic bile acids uptake, such as *Slco1a1*, *Slc10a1*, *Slco1a4*, *Slco2b1*, and *Slco1b2*, were not significantly affected by FGFR4 silencing in HFD-fed mice.

### The changes in liver and ileum transcriptome after FGFR4 KD

**Fig. 4:**
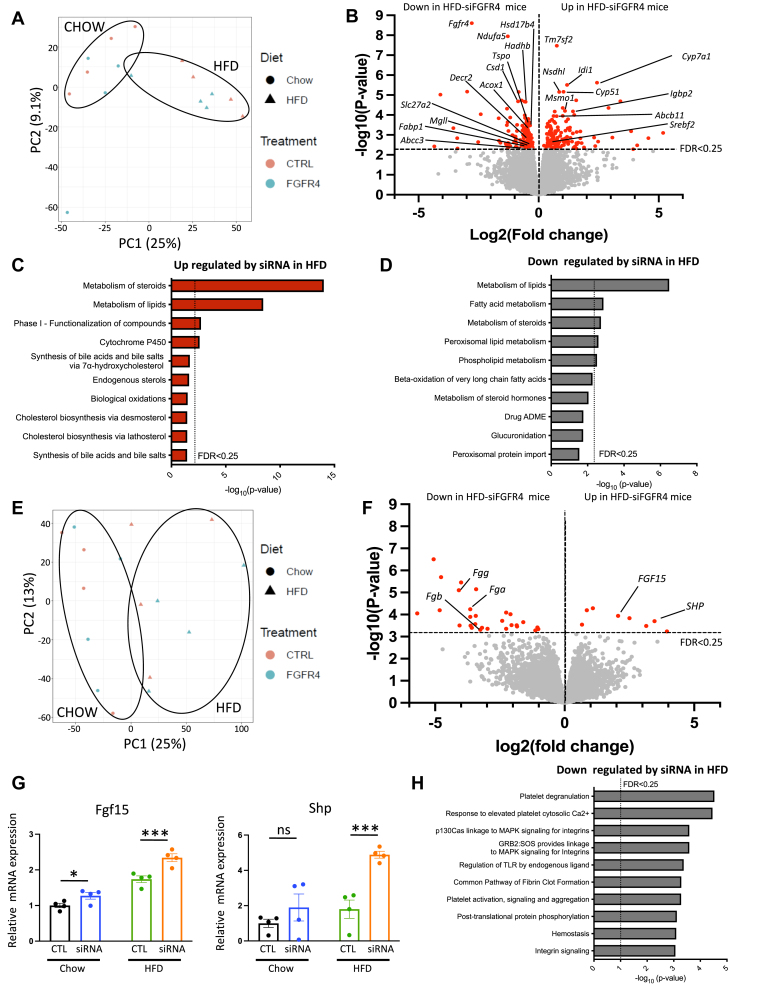
Characterization of gene expression changes induced by liver FGFR4 silencing in mice on HFD. Principal component analysis (PCA) (A) and volcano plot (B) of liver RNA-sequencing data from mice on HFD receiving either the saline solution or the siRNA targeting FGFR4. Most significant pathway upregulated (C) and downregulated (D) by liver FGFR4 silencing in HFD mice. Principal component analysis (PCA) (E) and volcano plot (F) of ileum RNA-sequencing data from mice on HFD receiving either the saline solution or the siRNA targeting FGFR4. G: Measure of *fgf15* and *Shp* gene expression in ileal biopsies from mice on HFD. Data are expressed as mean ± SEM and were normalized by the control condition in chow diet mice (n = 4, ns = non-significant, ∗ *P* < 0.05; ∗∗∗ *P* < 0.001, moderated *t* test). H: Most significant pathway downregulated by liver FGFR4 silencing in the ileum of HFD mice. FGFR, fibroblast growth factor receptor; HFD, high-fat diet.

Utilizing RNA-seq, we have performed a comprehensive assessment of global gene expression in liver and ileum of chow and HFD-fed mice treated with and without FGFR4 siRNA. The principal components analysis of the RNA-seq data from the liver revealed a clear separation between chow and HFD, but there was no effect of FGFR4 siRNA treatment on global gene expression (Fig. 4A). Among 12,870 identified genes, the expression of 274 genes was significantly altered (*P*-value < 0.05 FDR < 0.25) by silencing of FGFR4 in the liver of mice on HFD (Fig. 4B). The most upregulated gene in response to FGFR4 silencing in mice on an HFD was the transmembrane 7, superfamily member 2 (*Tmf7s2*) gene, also known as C-14 sterol reductase, involved in cholesterol synthesis. Other genes regulating cholesterol de novo synthesis such as *Srebf2, Abcb11, Idi1, Nsdhl, Cyp51*, and *Msmo1* were also upregulated in FGFR4 KD group (Figs. 4B, D and S4E). Consistent with an increase in bile acid synthesis and our qPCR results, the second most significantly upregulated gene induced by FGFR4 KD was *Cyp7A1*, the rate-limited enzyme of bile acid synthesis. In line with increased expression of genes mediating cholesterol and bile acid synthesis, pathway analysis reveals that cholesterol biosynthesis, endogenous sterol, and cytochrome P450 pathways were among the most upregulated processes with FGFR4 silencing (Fig. 4C). Moreover, *Igfbp2,* an emerging target for insulin resistance and liver steatosis, was increased with FGFR4 KD. As expected, *Fgfr4* was the most downregulated gene (Fig. 4B). Along with *Fgfr4*, genes involved in lipid metabolism such as *Fabp1*, *Hadhb*, *Tspo*, *Abcc3*, and *Mgll* were decreased. The mediators of mitochondrial function such as *Csd1, Ndufa5* and genes regulating peroxisomal lipid metabolism and fatty acid oxidation such as *Acox 1*, *Decr2, Slc27a2*, and *Hsd17b4* were suppressed in accordance with the downregulation of the peroxisomal lipid metabolism pathway (Fig. 4D). Interestingly, *Srebp1c*, the main regulators of lipid biosynthesis and its target genes, *Acaca*, *Fasn*, and *Scd1* were not significantly affected by FGFR4 KD in mice on HFD. Altogether, RNA-seq analysis of liver homogenates showed that FGFR4 silencing is associated with increased expression of genes involved in bile acid and cholesterol biosynthesis, while the expression of genes involved in lipid metabolism and fatty acid β-oxidation were decreased. Interestingly, the genes affected by hepatic FGFR4 KD are starkly different between chow- and HFD-fed mice (supplemental Fig. S6A). Thus, only one common gene, *Fgfr4,* was downregulated in both chow and HFD, whereas no common gene was upregulated in both chow and HFD (supplemental Fig. S3A, B).

Similar to the liver, the principal components analysis of the RNA-seq data from the ileum did not reveal a clear separation between the samples based on FGFR4 KD (Fig. 4E). Among the 15,794 identified genes in the ileum, the expression of only 37 genes was significantly altered (*P*-value < 0.05, FDR < 0.25) by the silencing of hepatic FGFR4 in mice on HFD. Volcano plot analysis showed that among the most significantly upregulated genes in the ileum of mice on HFD treated with FGFR4 siRNA were *Fgf15* and *Shp*, the main FXR targets induced by intestinal bile acids (Fig. 4F, G). This was confirmed by qPCR expression of *Fgf15* in the intestine (supplemental Fig. S5A). Circulating levels of FGF15 were stable in mice on chow diet, regardless of FGFR4 siRNA treatment and were not affected by an HFD (supplemental Fig. S5B). However, in mice on an HFD, FGFR4 silencing tended to increase circulating FGF15 levels in agreement with mRNA levels. In addition to *Fgf15* and *Shp,* the expression of other FXR targets in the intestine, *Glp1* and *Slc10a2*, was altered. Indeed, the expression of *Glp1* was strongly increased by FGFR4 KD in mice on an HFD (supplemental Fig. S5D). This is in agreement with the effect of bile acids on GLP1 secretion, via the activation of FXR, described in previous studies (29, 30). The pathway mediating bile acid intestinal transport including genes such as *Slc10a2* was repressed in mice on HFD with liver-specific FGFR4 KD (Fig. 3G). This was confirmed by qPCR quantification of *Slc10a2* expression (supplemental Fig. S5E). By comparison, *Fxr* expression and its target genes such as *Shp* and *Cyp8b1* were not significantly changed in the liver following FGFR4 silencing (supplemental Figs. S3A and S5F). Fibrinogen genes such as *Fgg*, *Fgb*, and *Fga,* involved in platelet activation and aggregation and integrins signaling, were significantly lower in FGFR4 KD mice (Fig. 4F, H). Taken together, following hepatic FGFR4 silencing, there was an increase of genes involved in FXR-FGF15 axis in the ileum and a repression of genes involved in bile acids absorption and inflammation. Interestingly, in mice on chow diet, there were no significantly altered genes by FGFR4 silencing. This is similar to gene expression in the liver of chow-fed mice where the only downregulated gene was FGFR4 (supplemental Fig. S6C).

### Silencing FGFR4 in the liver or primary hepatocytes increases IRS1 expression but decreases ERK phosphorylation

**Fig. 5:**
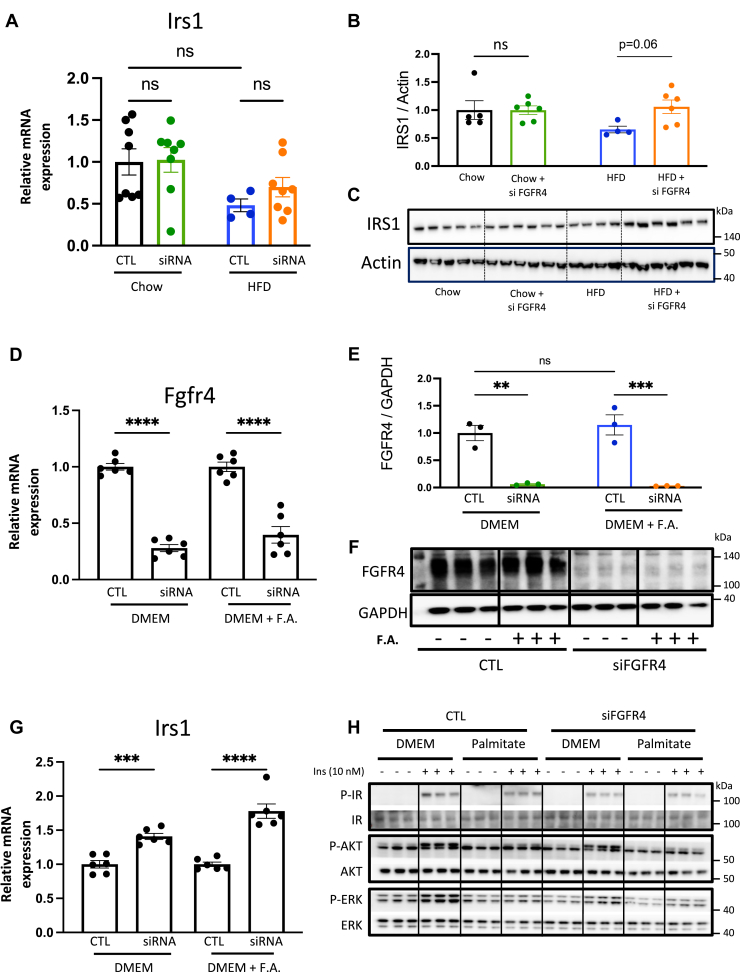
FGFR4 KD increases hepatic IRS1 expression. Measure of *Irs1* gene expression (A) and protein levels (B) in mice liver. Data are expressed as mean ± SEM (n = 4–8, two-way ANOVA followed by a Tukey’s multiple comparisons test). Data were normalized by the control condition in chow diet mice. C: Immunoblotting of IRS1 from liver biopsies. Measure of *Fgfr4* gene expression (D) and protein levels (E) and *Irs1* (G) gene expression in primary hepatocytes treated with 1000 ng/ml of an siRNA or a placebo in the presence or not of a mix of oleic acid and palmitic acid. F: Immunoblotting of FGFR4 from primary hepatocytes. H: Immunoblot of IR, AKT, and ERK phosphorylation in primary hepatocytes after insulin (10 nM) stimulation in the presence or not of palmitic acid. Data are expressed as mean ± SEM (n = 3–6, ns = non-significant, ∗∗ *P* < 0.01; ∗∗∗ *P* < 0.001; ∗∗∗∗*P* < 0.0001, two-way ANOVA followed by a Tukey’s multiple comparisons test). Data of each FGFR4 group were normalized by their own control for the figure D and G. FGFR, fibroblast growth factor receptor; IR, insulin receptor; KD, knockdown.

Bile acid treatment of diabetic mice has been shown to improve insulin resistance. To investigate the underlying mechanism behind improved insulin sensitivity following FGFR4 KD in mice on HFD, we interrogated insulin signaling pathway. *Irs1* expression in the liver was not changed by the hepatic FGFR4 silencing in mice on chow diet (Fig. 5A). On an HFD, hepatic *Irs1* expression was reduced by 50% and this was partially restored with FGFR4 KD*.* Consistent with this, protein level of IRS1 in the liver of mice on chow diet was not changed following FGFR4 siRNA treatment (Fig. 5B, C). Animals on HFD showed a trend to have decreased IRS1, while FGFR4 KD significantly increased hepatic IRS1 in these mice. To assess if increased *Irs1* was a direct consequence of FGFR4 silencing, or enterohepatic circulation of bile acids, mouse primary hepatocytes were treated with the FGFR4 siRNA in the presence or absence of free fatty acids. Primary hepatocytes treated with FGFR4 siRNA exhibited a strong decrease in *Fgfr4* expression by 72% in cells incubated without fatty acids and by 60% in the presence of fatty acids (Fig. 5D). Consistent with lower gene expression, FGFR4 protein was robustly decreased in cells treated with FGFR4 siRNA (Fig. 5E, F). Interestingly, *Irs1* expression was significantly increased by 30% following FGFR4 silencing in primary hepatocytes and by 45% after fatty acid addition (Fig. 5G). Since *Igfbp2* is one of the top upregulated genes in the liver by FGFR4 silencing, we assessed the consequence of FGFR4 KD on *Igfbp2* expression in vivo and in vitro. In chow-fed mice, hepatic *Igfbp2* expression tended to be increased by FGFR4 silencing (supplemental Fig. S7A). Interestingly, while HFD significantly decreases *Igfbp2* expression by 77%, silencing of FGFR4 restored its expression similar to the level seen in chow-fed control mice. However, neither FGFR4 silencing nor the addition of fatty acids to primary hepatocyte altered *Igfbp2* gene expression (supplemental Fig. S7B). To assess the consequence of FGFR4 KD on insulin signaling in vitro, IR, AKT, and ERK phosphorylation was measured after insulin stimulation (Fig. 5H). In line with previous works (31, 32), cells incubated with palmitate exhibit a reduction of phosphorylation for the IR, AKT, and ERK. Despite higher IRS1 mRNA, IR and AKT phosphorylation was not improved by FGFR4 silencing (Fig. 5H). On the other hand, FGFR4 KD profoundly decreased ERK phosphorylation consistent with a known function of FGFR4 to regulate cell growth (33). In summary, KD of FGFR4 in vivo and in vitro was associated with increased IRS1 mRNA and protein, suggesting a cell autonomous regulation. However, insulin signaling was not improved in vitro following FGFR4 KO, indicating that improved insulin signaling and *Igfbp2* expression in vivo were likely a consequence of improved hepatic steatosis in these mice.

## Discussion

In this study, we have investigated the role of the liver-specific FGFR4 silencing on hepatic steatosis, bile acid synthesis, and insulin resistance. A KD of FGFR4 has only a modest effect in mice on a chow diet. In contrast, a loss of hepatic FGFR4 in mice on HFD improves liver weight, hepatic triglycerides, and NAFLD activity score. This is associated with improved serum insulin, HOMA-IR measure of insulin resistance, and whole-body insulin sensitivity. Mechanistically, FGFR4 KD in mice on an HFD increases *Cyp7a1* expression and total circulating bile acid pool. This was associated with increased hepatic concentration of primary and secondary bile acids. Again, the changes in bile acid synthesis following FGFR4 KD were not observed in mice on a chow diet. RNA-seq analysis in the liver confirmed that *Cyp7a1* was one of the most upregulated genes following FGFR4 KD in mice on an HFD. Furthermore, there was a profound upregulation of cholesterol synthesis pathway, in agreement with lower hepatic cholesterol following FGFR4 KD. Lastly, RNA-seq analysis confirmed upregulation of insulin signaling pathway, especially *Irs1* and *Igfbp2* with FGFR4 KD. In the intestine, hepatic FGFR4 KD induced upregulation of *Shp* and *Fgf15* consistent with enhanced FXR signaling induced by bile acids. *In vitro*, silencing of FGFR4 in primary mouse hepatocytes resulted in cell autonomous upregulation of IRS1, but not in an improvement of insulin signaling, which is likely mediated by a decrease in steatosis. Taken together, FGFR4 silencing increased bile acid synthesis in mice on an HFD, but not on a chow diet. This is associated with improved NAFLD activity score and insulin resistance, implicating hepatic FGFR4 as an interesting target for the management of NAFLD.

One of the most intriguing observations following hepatic FGFR4 silencing is the improvement in liver steatosis induced by HFD feeding. Despite the modest effect of FGFR4 KD on weight gain, liver weight was significantly decreased in these mice. In agreement with liver size, hepatic triglycerides were elevated in mice on HFD and restored to normal following hepatic FGFR4 silencing. Lipid accumulation in the liver is a product of hepatic de novo lipogenesis, dietary lipid uptake in the liver, lipolysis of adipose tissue, hepatic fatty acid oxidation, and lipid secretion as VLDL (34, 35). The expression of genes regulating fatty acid synthesis is not affected by FGFR4 KD. *Srebp1c*, a master regulator of fatty acids synthesis, and its target genes *Scd1, Acaca*, *and Fasn* are not significantly altered. However, the expression of *Fabp1,* a major mediator of hepatic fatty acid uptake, was significantly decreased in FGFR4 KD mice on an HFD. An increase in fatty acid beta oxidation may ameliorate liver steatosis (34). Pathway analysis from RNA-seq data reveals that mitochondrial beta-oxidation pathway is reduced by FGFR4 KD in mice on HFD. Indeed, expression of *Slc27a2*, *Decr2, Hadhb*, and *Acox1* were strongly repressed in these mice. Lipids can be secreted out of the liver as bile acids (36). Bile acids are produced by the hepatocytes and secreted into bile canaliculi through the bile salt export pump (*Abcb11*). HFD has been previously described to decrease *Abcb11* gene expression, thus reducing bile acids secretion (37). Hepatic KD of FGFR4 restores *Abcb11* expression in the liver of mice on HFD, suggesting that bile acids efflux from the liver to the bile canaliculi may be increased in these mice. Indeed, overexpression of *Abcb11* in the liver is sufficient to decrease HFD-induced liver steatosis by increasing lipid secretion into the bile (36). In agreement with increased expression of genes involved in bile acid secretion, we observed an increase in the expression of *Cyp7a1*, a major gene regulating bile acid synthesis. Furthermore, primary and secondary bile acids were increased, while serum cholesterol is decreased, following a KD of FGFR4. Thus, increased bile acid synthesis and lipid secretion into the bile may account, in part, for a decrease in steatosis with FGFR4 KD in mice on HFD. Previous studies have shown that a whole body FGFR4 KO in mice on HFD improves liver steatosis (21, 38). What our study adds is that liver-specific FGFR4 KD is sufficient to induce this effect.

An interesting consequence of hepatic FGFR4 KD is a strong improvement of insulin sensitivity in mice fed an HFD. The role of hepatic FGFR4 in glucose metabolism and insulin sensitivity is inconsistent in the literature. A whole-body knockout of FGFR4 in mice on chow diet induced features of metabolic syndrome including increased adiposity, hyperlipidemia, and insulin resistance (21). However, a whole-body KO of FGFR4 in mice on HFD improved hepatic lipid accumulation, but not dyslipidemia or insulin resistance (21). In contrary, Hongfei Ge *et al.* has shown that whole body KO for FGFR4 in mice on HFD improves glucose tolerance and insulin sensibility (20). Similar to our study, peripheral silencing of FGFR4 via an antisense oligonucleotide in diet-induced obese mice has been shown to improve insulin sensitivity (38). In our study, the beneficial effect of hepatic FGFR4 silencing on glucose metabolism is primarily evident in terms of insulin sensitivity. Interestingly, silencing of FGFR4 in mouse primary hepatocytes is sufficient to increase *Irs1* expression, suggesting that *Irs1* upregulation is a direct consequence of FGFR4 deficiency. Despite a robust increase of Irs1, insulin signaling in the primary hepatocytes was not increased following FGFR4 silencing. These results suggest that an improvement of insulin sensitivity, seen in vivo, is not a direct consequence of the FGFR4 hepatic loss but likely reflects an improvement in steatosis. Similarly, an increase in *Igfbp2* following FGFR4 KD is only observed in vivo*, but it is* unchanged in mouse primary hepatocyte after FGFR4 silencing. This suggests that *Igfbp2* expression may be regulated by circulating factors independently of the hepatic FGFR4 *signaling*. Indeed, IGFBP2 is increased in mice (39) and humans (40) following bariatric surgery, which increases circulating bile acids. IGFBP2 has emerged as an important mediator of insulin resistance (41) and metabolic health (42). Circulating levels of IGFBP2 are low in obese patients and in diet-induced obese mice (41). Moreover, low levels of IGFBP2 were described in patients with type 2 diabetes, while higher levels are associated with insulin sensitivity (43, 44). Taken together, our findings indicate that improvement in insulin sensitivity is not a direct consequence of hepatic FGFR4 silencing but may be mediated by circulating factors such as bile acids and gut hormones or be secondary to improved liver steatosis.

FGFR4, along with FXR, is a master regulator of CYP7A1, a key enzyme for bile acids synthesis (45). Both FXR and FGFR4 activation lead to the inhibition of *Cyp7a1* gene expression. Our data shows that hepatic FGFR4 silencing strongly increases *Cyp7a1* expression and therefore increases bile acids synthesis. This is only observed in obese mice on HFD, but not on chow diet, since HFD provides ample lipid precursors for bile acid synthesis. In our study, higher expression of Cyp7a1 is not associated with a significant decrease of body weight in mice on HFD. In contrast, Li *et al.* have shown that CYP7A1 transgenic mice are protected against body weight gain induced by HFD (46). We hypothesize that this disparity may be due to a more physiologic increase in *Cyp7a1* expression in our model and slow rise in Cyp7a1 expression, which was not evident at two weeks but was evident at 10 weeks of HFD feeding after the mice gained significant amount of weight. Since bile acids are mainly produced from cholesterol in the liver, it was not surprising to notice a decrease of circulating levels of cholesterol concomitant with an increase in bile acids synthesis. Indeed, several genes involved in cholesterol de novo synthesis are strongly increased in response to FGFR4 silencing. Thus, expression of *Tm7sf2*, *Idi1*, *Nsdhl*, *Cyp51*, and *Msmo1* were upregulated by FGFR4 KD. This suggests that hepatic cholesterol synthesis increases to provide substrate for bile acid production. Moreover, the expression of hepatic *Ldlr* is unchanged signifying that cholesterol uptake by the liver is not affected. Interestingly, studies have shown that a whole body FGFR4 KO does not change or even increase hypercholesterolemia, despite stimulation of bile acid synthesis (20, 21). The discrepancy between whole body and liver-specific FGFR4 KO implicates the importance of FXR stimulation in the intestine by increased bile acid secretion. Our data indicates that the effects of hepatic FGFR4 KD are predominant over hepatic FXR activation by bile acids to regulate bile acid production.

Once produced by the liver, bile acids are secreted into the intestinal lumen where they participate in dietary lipid and fat-soluble vitamin absorption. In the enterocytes, bile acids activate FXR leading to the secretion of the gut hormone FGF15/19. Indeed, we observe an increase in *Fgf15*, *Shp*, and *Glp1* expression in the intestines of the mice following silencing of hepatic FGFR4. The activation of liver FGFR4 by FGF15/19 inhibits liver *Cyp7a1* gene expression and thus reduces bile acids synthesis. In our study, a lack of liver FGFR4 interrupts this negative feedback mechanism leading to a chronic increase of hepatic bile acids. Chenodeoxycholic acid and tauro-chenodeoxycholic acid are the most potent bile acids to activate FXR (47). While circulating levels of CDCA are not significantly changed by hepatic FGFR4 silencing, its tauro-conjugated form is strongly increased suggesting that the intestinal FXR is activated. In line with a potential FXR activation, the expression of the main FXR target genes in the ileum is significantly elevated. *Shp* and *Fgf15* are the main upregulated genes in the ileum in FGFR4 KD mice on HFD. On the contrary, gene expression of *Slc10a2*, negatively regulated by FXR, is reduced (*P*-value = 0.004, FDR = 0.57). Before reaching the liver to activate FGFR4, FGF15/19 may act locally. Indeed, the expression of FGFR4 increases along the small intestine with a very modest presence in the duodenum and jejunum and a higher abundance in the ileum and the colon (15). In caco2 cells, a colonic cell line, FGF19 treatment reduces *Slc10A2* gene expression via FGFR4-βKLOTHO activation (48). Jyoti *et al.* suggest that FXR may be a mediator of the action of FGF19 on enterocyte. The increase of FGF15 secretion correlates with the decrease of *Slc10a2* gene expression in the ileum suggests that FGF15 may act via an autocrine pathway on the intestinal cells and induce FXR activation in synergy with the action of the bile acids. Taken together, our results suggest that intestinal FXR activation may be sufficient to improve hepatic lipid homeostasis and insulin signaling.

Targeting FXR is one of the strategies currently explored in clinical trials to treat NAFLD (49). While activation of the liver FXR is postulated to attenuate liver steatosis, modulation of the intestinal FXR is becoming an emerging strategy for the treatment of NAFLD (29, 50, 51). In our model, only the intestinal FXR is activated since hepatic FXR target genes such as *Shp* and *Cyp8b1* are not affected. This suggests that the improvement in liver steatosis and insulin sensitivity seen in FGFR4 KD mice are not dependent on hepatic FXR activation. On the other hand, intestinal FXR activation leading to the upregulation of FGF15 could mediate the reduction of hepatic lipid accumulation. Although the liver FGFR4 is silenced, the beneficial effects of FGF15/19 on liver steatosis may be due to its action via FGFR4 in the intestine or FGFR1 in the liver or adipose tissue. The absence of hepatic FGFR4 in the setting of increased circulating FGF15/19 provides several advantages. First, FGF15/19 activation of FGFR4 has been associated with abnormal hepatocytes proliferation and increased risk of liver cancer (52). We show that FGFR4 KD in primary hepatocytes decreases ERK phosphorylation, which should ameliorate this risk. Moreover, activation of the intestinal FXR-FGF15/19 axis can be associated with undesirable increase in circulating cholesterol levels, due to the repression of hepatic bile acid production via Cyp7a1 (53). In our model, selective silencing of hepatic FGFR4 leads to a lack of CYP7A1 feedback inhibition. This results in an ongoing increase in hepatic bile acids and a decrease of plasma cholesterol. Thus, silencing FGFR4 leading to activation of the intestinal FXR-FGF15/19 axis allows for advantageous effects of FGF15/19 while avoiding the risk of hypercholesterolemia and hepatic carcinogenesis.

In conclusion, we show that a liver FGFR4 KD improves liver steatosis by increasing hepatic bile acid synthesis. Moreover, liver FGFR4 silencing improves insulin sensitivity and reduces circulating cholesterol levels. These improvements seem to be independent of the liver FXR signaling but may be mediated via intestinal FXR. Activation of the intestinal FXR likely leads to systemic FGF15-mediated metabolic benefits, resulting in improved liver steatosis and hepatic insulin resistance. Hepatic silencing of FGFR4 leading to activation of the intestinal FXR-FGF15/19 axis could be a promising strategy to treat metabolic disorders induced by HFD.

## Data Availability

The data supporting this work are available upon reasonable request from the corresponding author. RNAseq data from the liver (GSE222171) and intestine (GSE222499) have been deposited to GEO, a public reportory.

## Supplemental data

This article contains supplemental data.

## Conflict of Interest

F. T. and K. F. are employees of Alnylam Pharmaceuticals. All other authors declare no conflicts of interest with the contents of this article.

## References

[1] Browning J.D., Horton J.D. (2004). Molecular mediators of hepatic steatosis and liver injury. J. Clin. Invest..

[2] Mitra S., De A., Chowdhury A. (2020). Epidemiology of non-alcoholic and alcoholic fatty liver diseases. Transl Gastroenterol. Hepatol..

[3] Noureddin M., Vipani A., Bresee C., Todo T., Kim I.K., Alkhouri N. (2018). NASH Leading cause of liver transplant in women: updated analysis of indications for liver transplant and ethnic and gender variances. Am. J. Gastroenterol..

[4] Younossi Z.M., Golabi P., de Avila L., Paik J.M., Srishord M., Fukui N. (2019). The global epidemiology of NAFLD and NASH in patients with type 2 diabetes: a systematic review and meta-analysis. J. Hepatol..

[5] Younossi Z.M., Ratziu V., Loomba R., Rinella M., Anstee Q.M., Goodman Z. (2019). Obeticholic acid for the treatment of non-alcoholic steatohepatitis: interim analysis from a multicentre, randomised, placebo-controlled phase 3 trial. Lancet.

[6] Myant N.B., Mitropoulos K.A. (1977). Cholesterol 7 alpha-hydroxylase. J. Lipid Res..

[7] Siddiqui M.S., Van Natta M.L., Connelly M.A., Vuppalanchi R., Neuschwander-Tetri B.A., Tonascia J. (2020). Impact of obeticholic acid on the lipoprotein profile in patients with non-alcoholic steatohepatitis. J. Hepatol..

[8] Xie Y., Su N., Yang J., Tan Q., Huang S., Jin M. (2020). FGF/FGFR signaling in health and disease. Signal. Transduct Target Ther..

[9] Dionne C.A., Crumley G., Bellot F., Kaplow J.M., Searfoss G. (1990). Cloning and expression of two distinct high-affinity receptors cross-reacting with acidic and basic fibroblast growth factors. EMBO J..

[10] Partanen J., Mäkelä T.P., Alitalo R., Lehväslaiho H., Alitalo K. (1990). Putative tyrosine kinases expressed in K-562 human leukemia cells. Proc. Natl. Acad. Sci. U. S. A..

[11] Eisemann A., Ahn J.A., Graziani G., Tronick S.R., Ron D. (1991). Alternative splicing generates at least five different isoforms of the human basic-FGF receptor. Oncogene.

[12] Hou J.Z., Kan M.K., McKeehan K., McBride G., Adams P., McKeehan W.L. (1991). Fibroblast growth factor receptors from liver vary in three structural domains. Science.

[13] Miki T., Bottaro D.P., Fleming T.P., Smith C.L., Burgess W.H., Chan A.M. (1992). Determination of ligand-binding specificity by alternative splicing: two distinct growth factor receptors encoded by a single gene. Proc. Natl. Acad. Sci. U. S. A..

[14] Dai S., Zhou Z., Chen Z., Xu G., Chen Y. (2019). Fibroblast Growth Factor Receptors (FGFRs): structures and small molecule inhibitors. Cells.

[15] Fon Tacer K., Bookout A.L., Ding X., Kurosu H., John G.B., Wang L. (2010). Research resource: comprehensive expression atlas of the fibroblast growth factor system in adult mouse. Mol. Endocrinol..

[16] Chiang J.Y., Kimmel R., Weinberger C., Stroup D. (2000). Farnesoid X receptor responds to bile acids and represses cholesterol 7alpha-hydroxylase gene (CYP7A1) transcription. J. Biol. Chem..

[17] Song K.H., Li T., Owsley E., Strom S., Chiang J.Y. (2009). Bile acids activate fibroblast growth factor 19 signaling in human hepatocytes to inhibit cholesterol 7alpha-hydroxylase gene expression. Hepatology.

[18] Hofmann A.F. (1999). The continuing importance of bile acids in liver and intestinal disease. Arch. Intern. Med..

[19] Chiang J.Y., Pathak P., Liu H., Donepudi A., Ferrell J., Boehme S. (2017). Intestinal farnesoid X receptor and takeda G protein couple receptor 5 signaling in metabolic regulation. Dig. Dis..

[20] Ge H., Zhang J., Gong Y., Gupte J., Ye J., Weiszmann J. (2014). Fibroblast growth factor receptor 4 (FGFR4) deficiency improves insulin resistance and glucose metabolism under diet-induced obesity conditions. J. Biol. Chem..

[21] Huang X., Yang C., Luo Y., Jin C., Wang F., McKeehan W.L. (2007). FGFR4 prevents hyperlipidemia and insulin resistance but underlies high-fat diet induced fatty liver. Diabetes.

[22] Wu X., Ge H., Lemon B., Weiszmann J., Gupte J., Hawkins N. (2009). Selective activation of FGFR4 by an FGF19 variant does not improve glucose metabolism in ob/ob mice. Proc. Natl. Acad. Sci. U. S. A..

[23] Matsuda S., Keiser K., Nair J.K., Charisse K., Manoharan R.M., Kretschmer P. (2015). siRNA conjugates carrying sequentially assembled trivalent N-acetylgalactosamine linked through nucleosides elicit robust gene silencing in vivo in hepatocytes. ACS Chem. Biol..

[24] Nair J.K., Willoughby J.L., Chan A., Charisse K., Alam M.R., Wang Q. (2014). Multivalent N-acetylgalactosamine-conjugated siRNA localizes in hepatocytes and elicits robust RNAi-mediated gene silencing. J. Am. Chem. Soc..

[25] D'Souza A.A., Devarajan P.V. (2015). Asialoglycoprotein receptor mediated hepatocyte targeting - strategies and applications. J. Control Release.

[26] Softic S., Gupta M.K., Wang G.X., Fujisaka S., O'Neill B.T., Rao T.N. (2017). Divergent effects of glucose and fructose on hepatic lipogenesis and insulin signaling. J. Clin. Invest..

[27] Softic S., Boucher J., Solheim M.H., Fujisaka S., Haering M.F. (2016). Lipodystrophy due to adipose tissue-specific insulin receptor knockout results in progressive NAFLD. Diabetes.

[28] Garcia-Martin R., Brandao B.B., Thomou T., Altindis E., Kahn C.R. (2022). Tissue differences in the exosomal/small extracellular vesicle proteome and their potential as indicators of altered tissue metabolism. Cell Rep..

[29] Pathak P., Xie C., Nichols R.G., Ferrell J.M., Boehme S., Krausz K.W. (2018). Intestine farnesoid X receptor agonist and the gut microbiota activate G-protein bile acid receptor-1 signaling to improve metabolism. Hepatology.

[30] Adrian T.E., Ballantyne G.H., Longo W.E., Bilchik A.J., Graham S., Basson M.D. (1993). Deoxycholate is an important releaser of peptide YY and enteroglucagon from the human colon. Gut.

[31] Ishii M., Maeda A., Tani S., Akagawa M. (2015). Palmitate induces insulin resistance in human HepG2 hepatocytes by enhancing ubiquitination and proteasomal degradation of key insulin signaling molecules. Arch. Biochem. Biophys..

[32] Ding Y., Xian X., Holland W.L., Tsai S., Herz J. (2016). Low-density lipoprotein receptor-related protein-1 protects against hepatic insulin resistance and hepatic steatosis. EBioMedicine.

[33] French D.M., Lin B.C., Wang M., Adams C., Shek T., Hötzel K. (2012). Targeting FGFR4 inhibits hepatocellular carcinoma in preclinical mouse models. PLoS One.

[34] Ipsen D.H., Lykkesfeldt J., Tveden-Nyborg P. (2018). Molecular mechanisms of hepatic lipid accumulation in non-alcoholic fatty liver disease. Cell Mol. Life Sci..

[35] Softic S., Cohen D.E., Kahn C.R. (2016). Role of dietary fructose and hepatic de novo lipogenesis in fatty liver disease. Dig. Dis. Sci..

[36] Figge A., Lammert F., Paigen B., Henkel A., Matern S., Korstanje R. (2004). Hepatic overexpression of murine Abcb11 increases hepatobiliary lipid secretion and reduces hepatic steatosis. J. Biol. Chem..

[37] Li S., Xu S., Zhao Y., Wang H., Feng J. (2020). Dietary betaine addition promotes hepatic cholesterol synthesis, bile acid conversion, and export in rats. Nutrients.

[38] Yu X.X., Watts L.M., Manchem V.P., Chakravarty K., Monia B.P., McCaleb M.L. (2013). Peripheral reduction of FGFR4 with antisense oligonucleotides increases metabolic rate and lowers adiposity in diet-induced obese mice. PLoS One.

[39] Faramia J., Hao Z., Mumphrey M.B., Townsend R.L., Miard S., Carreau A.M. (2021). IGFBP-2 partly mediates the early metabolic improvements caused by bariatric surgery. Cell Rep. Med..

[40] Li Z., Martin J., Poirier P., Caron-Cantin S.M., Hould F.S., Marceau S. (2012). Upregulation of plasma insulin-like growth factor binding protein 2 levels after biliopancreatic diversion in humans. Obesity (Silver Spring).

[41] Wheatcroft S.B., Kearney M.T., Shah A.M., Ezzat V.A., Miell J.R., Modo M. (2007). IGF-binding protein-2 protects against the development of obesity and insulin resistance. Diabetes.

[42] Boughanem H., Yubero-Serrano E.M., López-Miranda J., Tinahones F.J., Macias-Gonzalez M. (2021). Potential role of insulin growth-factor-binding protein 2 as therapeutic target for obesity-related insulin resistance. Int. J. Mol. Sci..

[43] Frystyk J., Skjaerbaek C., Vestbo E., Fisker S., Orskov H. (1999). Circulating levels of free insulin-like growth factors in obese subjects: the impact of type 2 diabetes. Diabetes Metab. Res. Rev..

[44] Yau S.W., Harcourt B.E., Kao K.T., Alexander E.J., Russo V.C., Werther G.A. (2018). Serum IGFBP-2 levels are associated with reduced insulin sensitivity in obese children. Clin. Obes..

[45] Holt J.A., Luo G., Billin A.N., Bisi J., McNeill Y.Y., Kozarsky K.F. (2003). Definition of a novel growth factor-dependent signal cascade for the suppression of bile acid biosynthesis. Genes Dev..

[46] Li T., Owsley E., Matozel M., Hsu P., Novak C.M., Chiang J.Y. (2010). Transgenic expression of cholesterol 7alpha-hydroxylase in the liver prevents high-fat diet-induced obesity and insulin resistance in mice. Hepatology.

[47] Lew J.L., Zhao A., Yu J., Huang L., De Pedro N., Peláez F. (2004). The farnesoid X receptor controls gene expression in a ligand- and promoter-selective fashion. J. Biol. Chem..

[48] Sinha J., Chen F., Miloh T., Burns R.C., Yu Z., Shneider B.L. (2008). beta-Klotho and FGF-15/19 inhibit the apical sodium-dependent bile acid transporter in enterocytes and cholangiocytes. Am. J. Physiol. Gastrointest. Liver Physiol..

[49] Trauner M., Fuchs C.D. (2022). Novel therapeutic targets for cholestatic and fatty liver disease. Gut.

[50] Jiang C., Xie C., Li F., Zhang L., Nichols R.G., Krausz K.W. (2015). Intestinal farnesoid X receptor signaling promotes nonalcoholic fatty liver disease. J. Clin. Invest..

[51] Clifford B.L., Sedgeman L.R., Williams K.J., Morand P., Cheng A., Jarrett K.E. (2021). FXR activation protects against NAFLD via bile-acid-dependent reductions in lipid absorption. Cell Metab..

[52] Raja A., Park I., Haq F., Ahn S.M. (2019). FGF19-FGFR4 signaling in hepatocellular carcinoma. Cells.

[53] Chávez-Talavera O., Tailleux A., Lefebvre P., Staels B. (2017). Bile acid control of metabolism and inflammation in obesity, type 2 diabetes, dyslipidemia, and nonalcoholic fatty liver disease. Gastroenterology.

