# Supplementary figures and images for: Liver-specific FGFR4 knockdown in mice on an HFD increases bile acid synthesis and improves hepatic steatosis
Source: J Lipid Res. 2022 Dec 29;64(2):100324. doi: 10.1016/j.jlr.2022.100324 (PMC9871743; doi:10.1016/j.jlr.2022.100324)

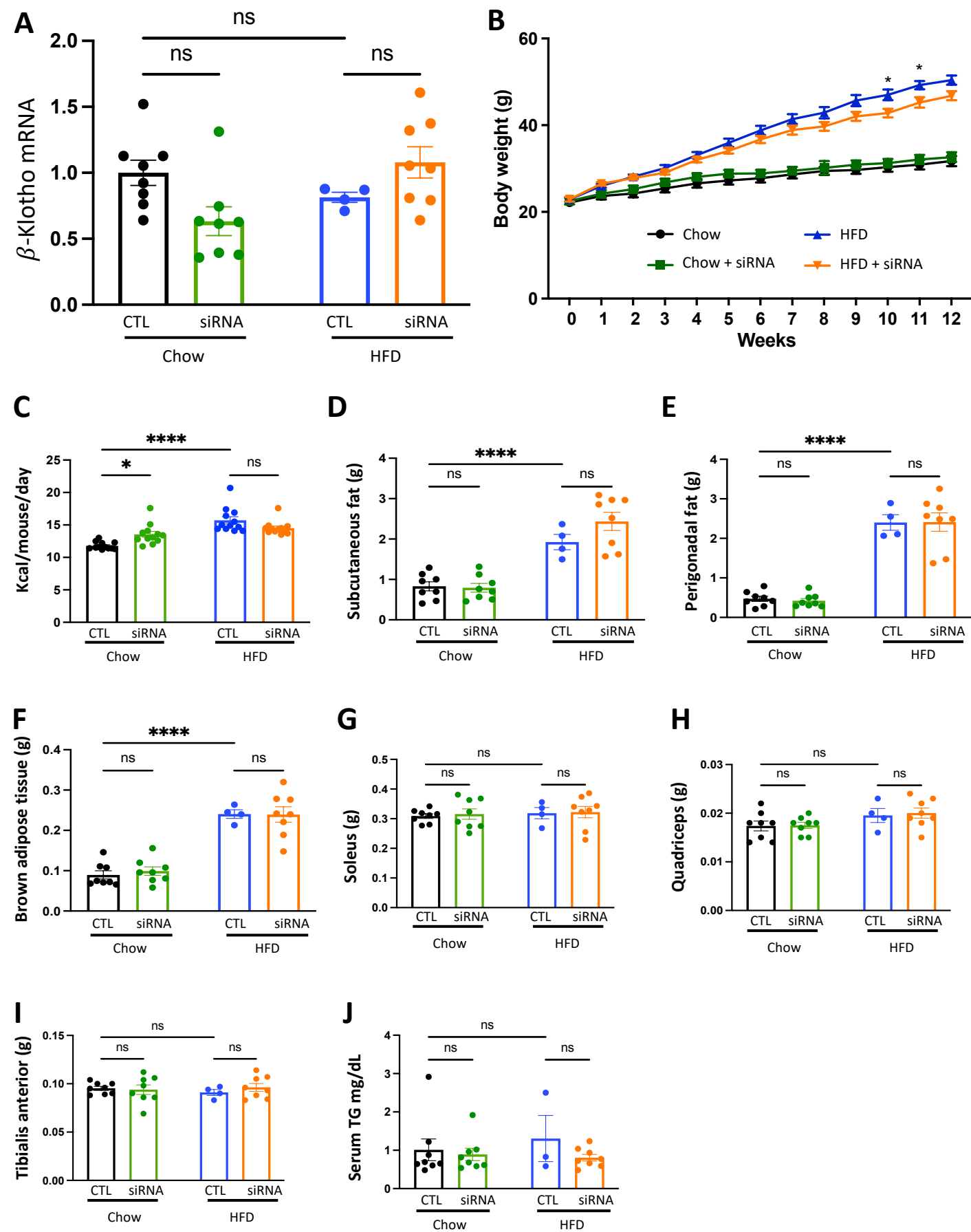

Supplement: Supplemental Fig 1 [file mmc1.pdf]

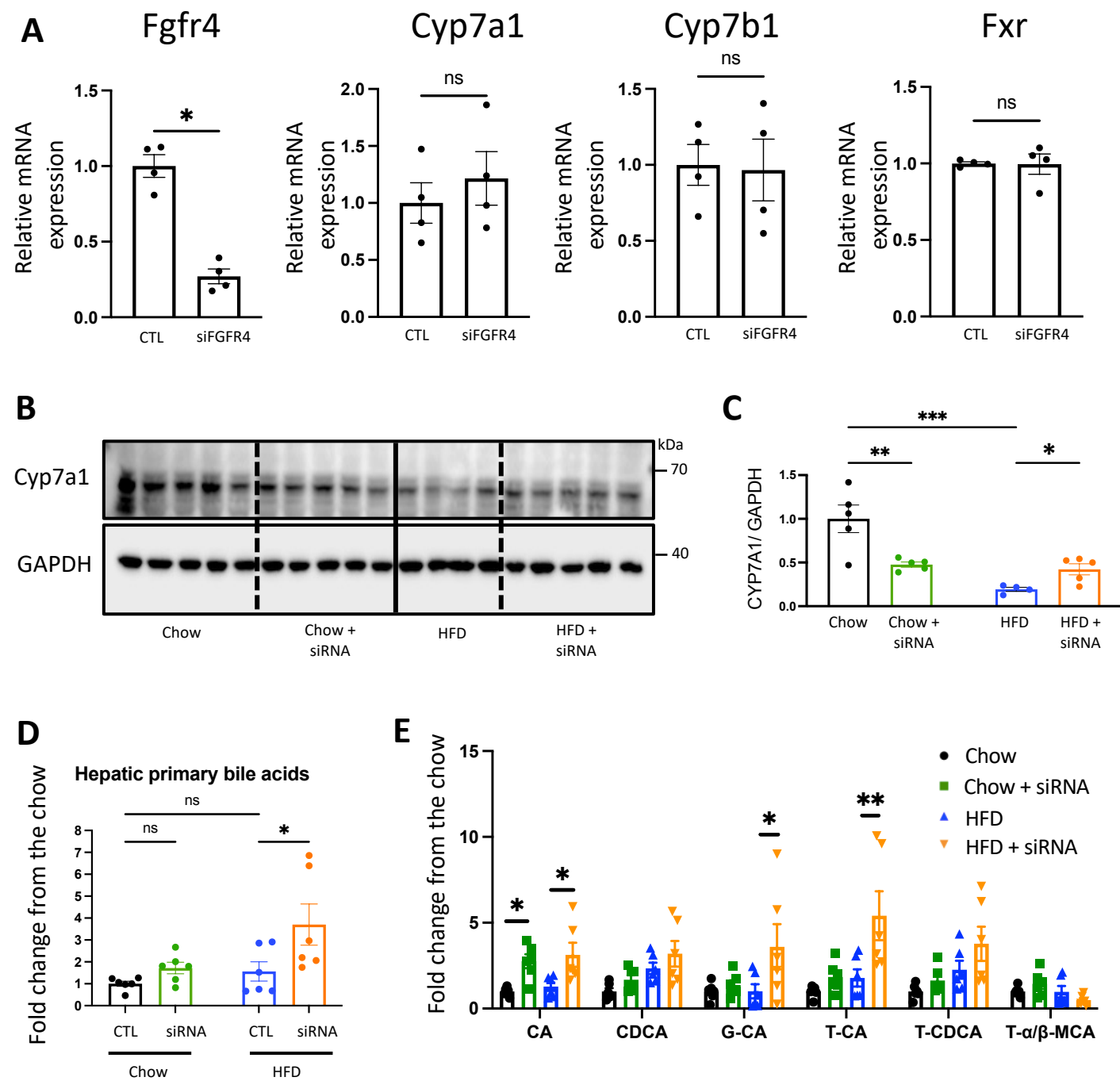

Supplement: Supplemental Fig 2 [file mmc2.pdf]

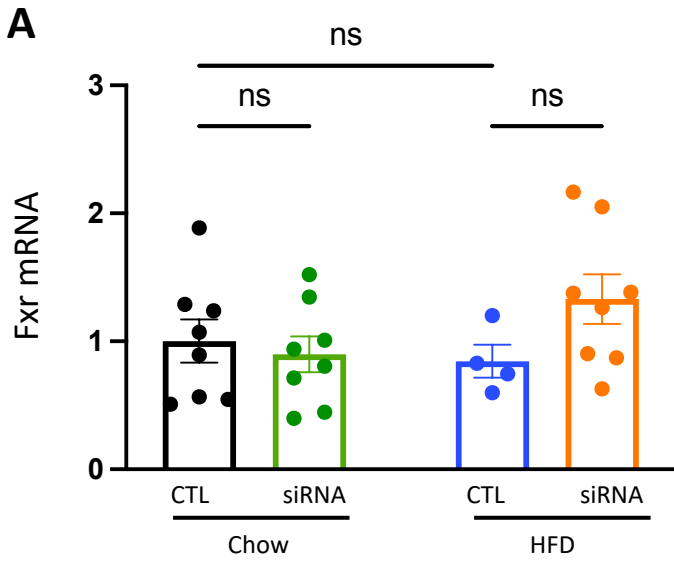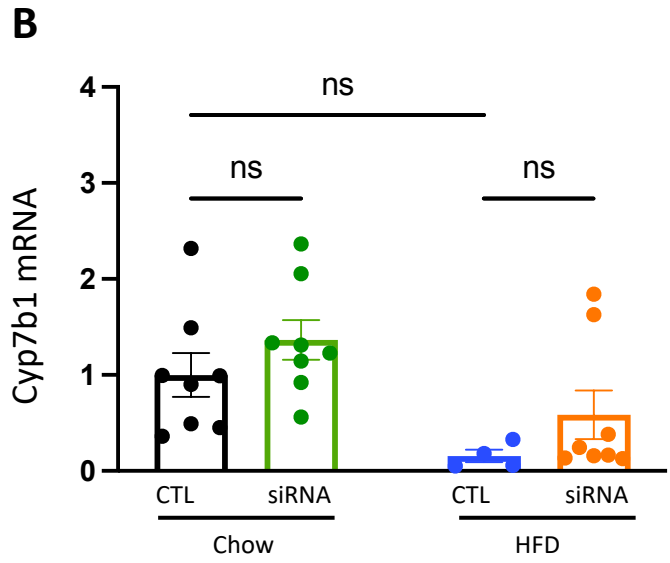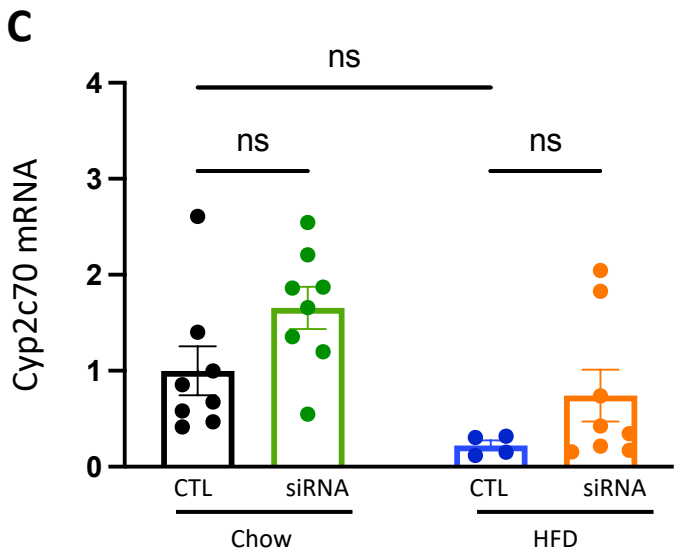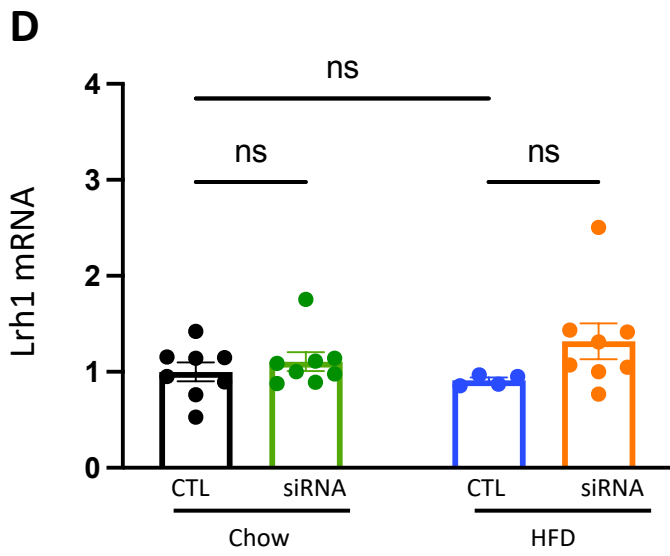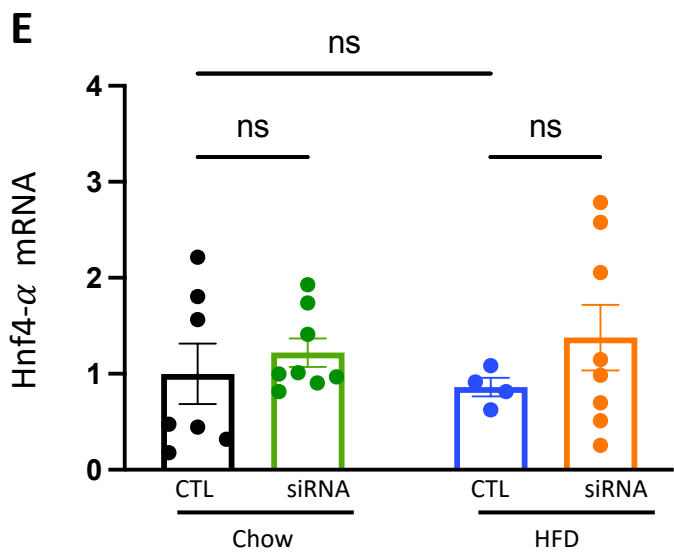

Supplement: Supplemental Fig 3 [file mmc3.pdf]

**A**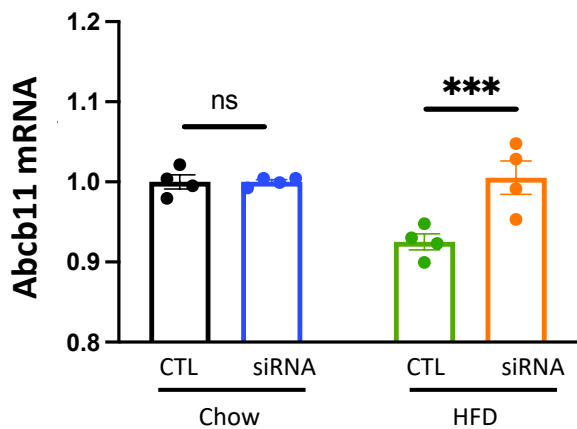**B**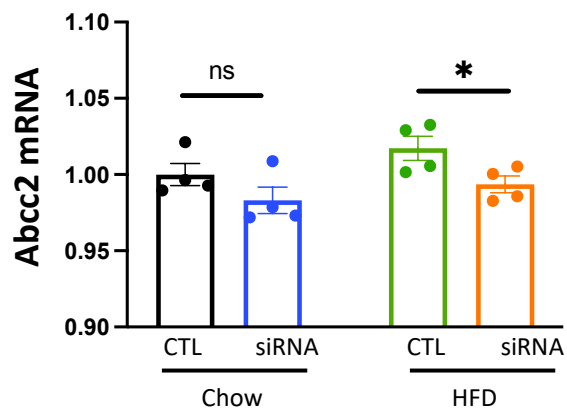**C**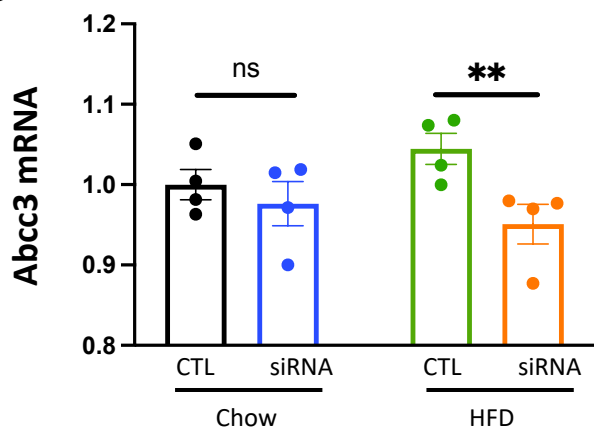**D**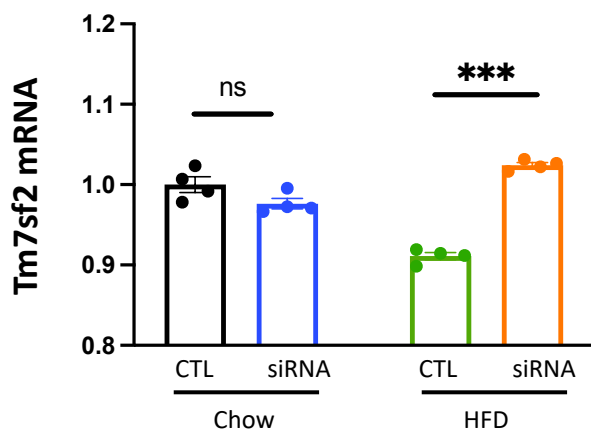**E**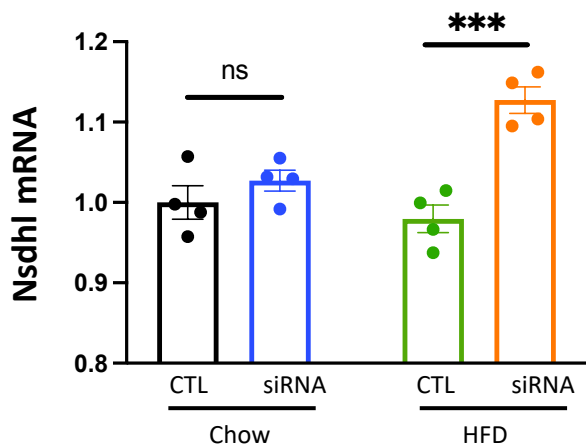**F**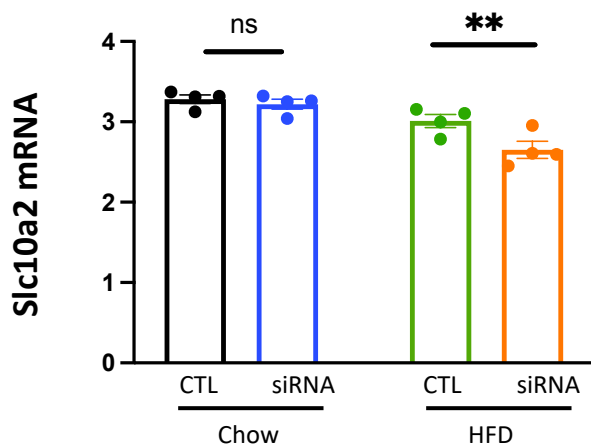

Supplement: Supplemental Fig 4 [file mmc4.pdf]

**A**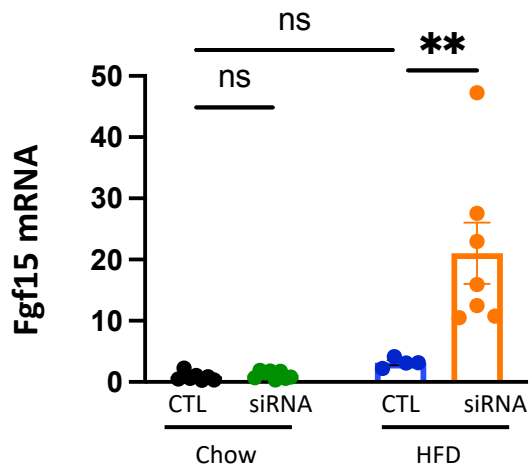**B**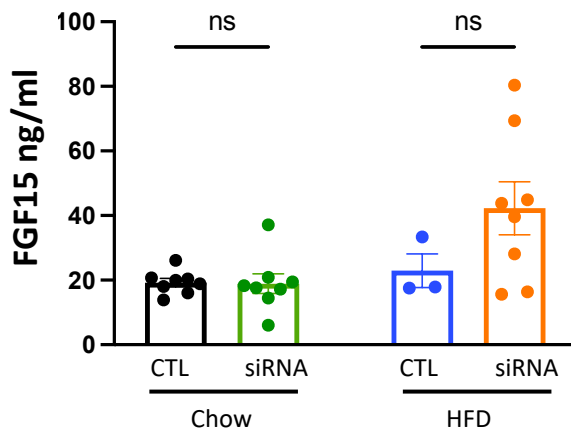**C**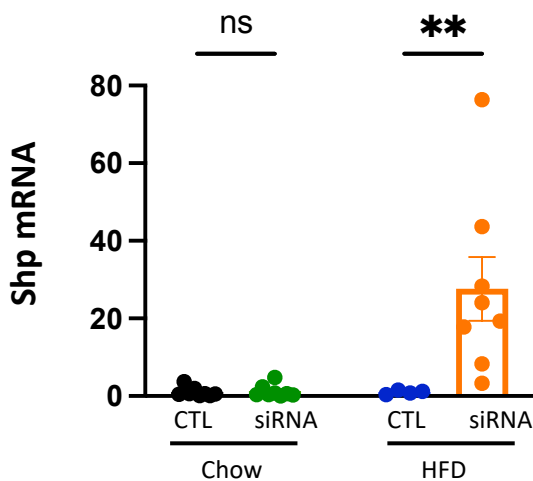**D**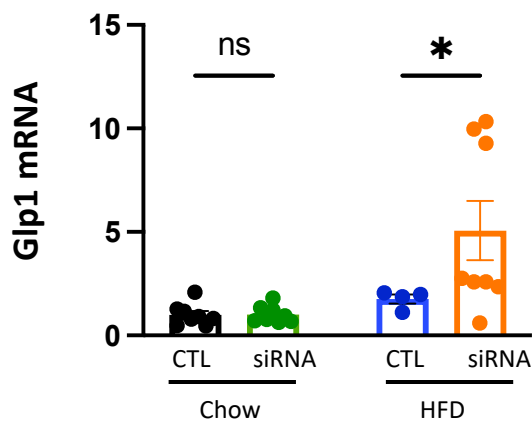**E**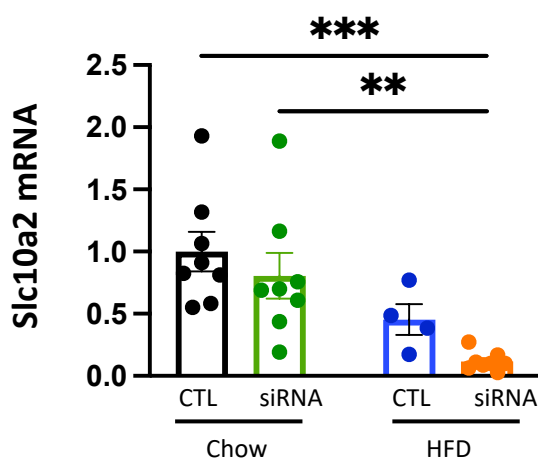**F**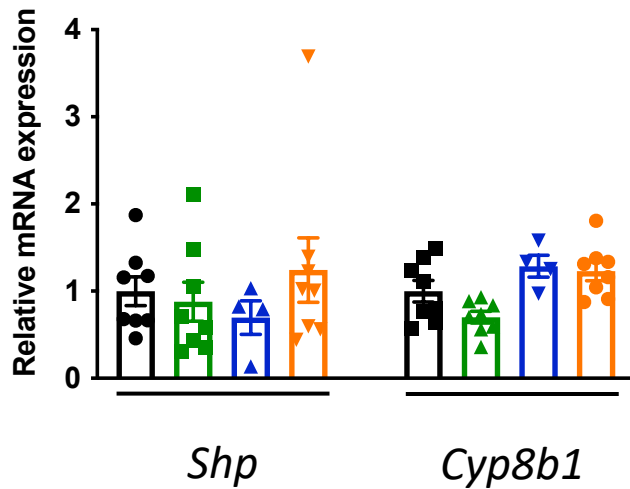

Supplement: Supplemental Fig 5 [file mmc5.pdf]

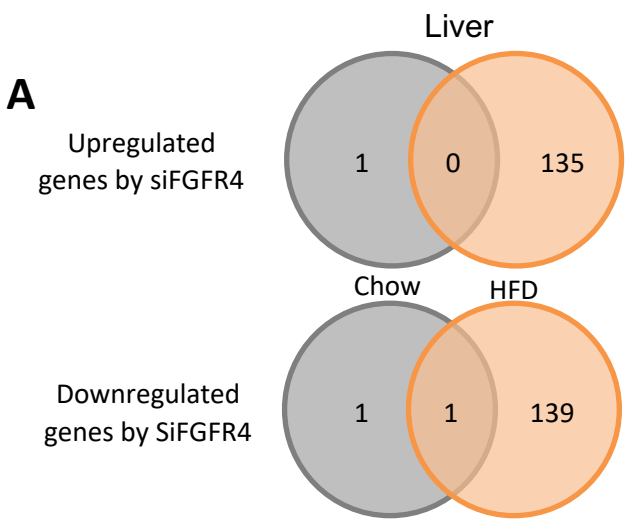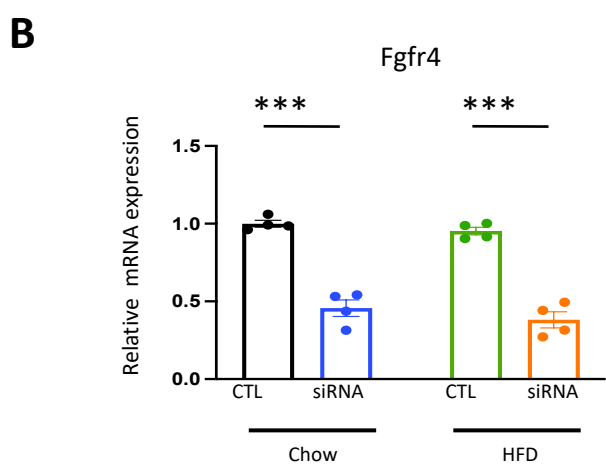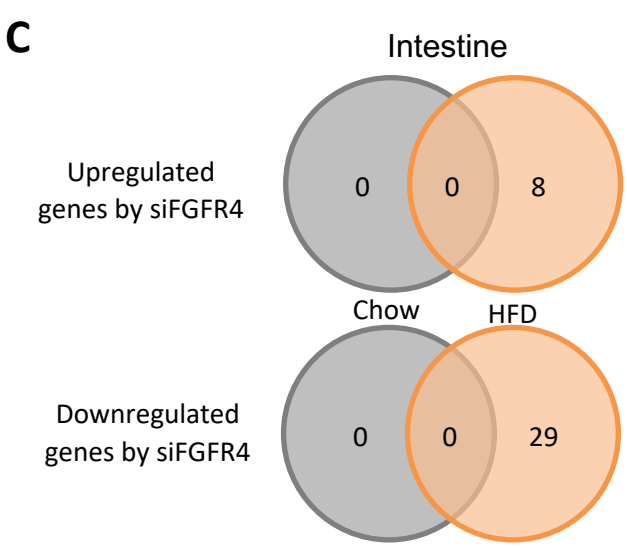

Supplement: Supplemental Fig 6 [file mmc6.pdf]

**A**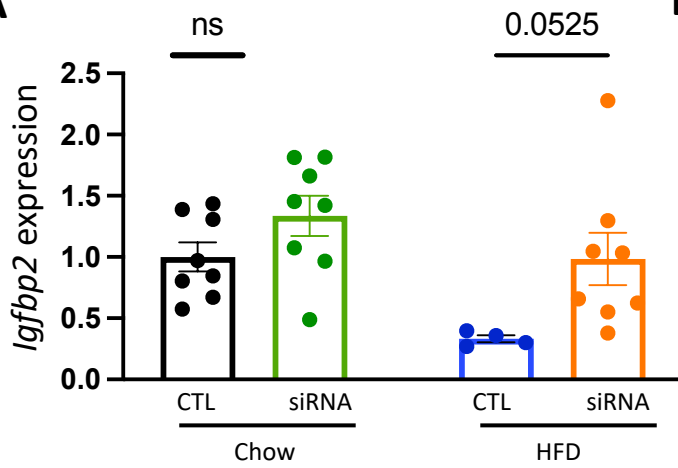**B**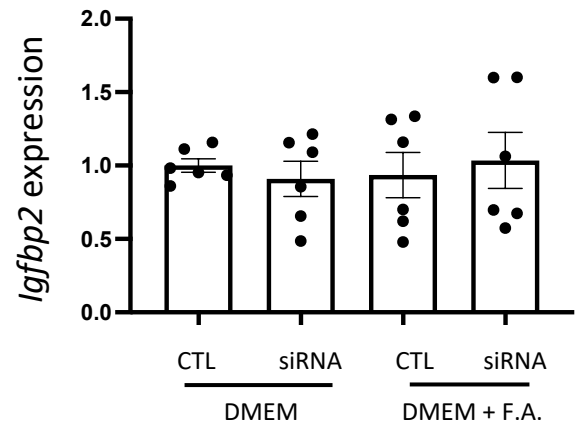

Supplement: Supplemental Fig 7 [file mmc7.pdf]
