# Supplementary material for: Liver-specific FGFR4 knockdown in mice on an HFD increases bile acid synthesis and improves hepatic steatosis
Source: J Lipid Res. 2022 Dec 29;64(2):100324. doi: 10.1016/j.jlr.2022.100324 (PMC9871743; doi:10.1016/j.jlr.2022.100324)
Supplement: Supplemental Table [file mmc8.docx]

**Supplementary Table 1**

| *Fgfr1* | Forward  Reverse | GCCAGACAACTTGCCGTATG  ATTTCCTTGTCGGTGGTATTAACTC |
| --- | --- | --- |
| *Fgfr2* | Forward  Reverse | CCCGGCCCTCCTTCA  GTTGGGAGATTTGGTATTTGGTT |
| *Fgfr3* | Forward  Reverse | TGGATCAGTGAGAATGTGGAGG  CCTATGAAATTGGTGGCTCGAC |
| *Fgfr4* | Forward  Reverse | CGCCAGCCTGTCACTATACAAA  CCAGAGGACCTCGACTCCAA |
| *Cyp7a1* | Forward  Reverse | GGGATTGCTGTGGTAGTGAGC  GGTATGGAATCAACCCGTTGTC |
| *Irs1* | Forward  Reverse | TGTCACCCAGTGGTAGTTGCTC  CTCTCAACAGGAGGTTTGGCATG |
| *Fgf15* | Forward  Reverse | ACGTCCTTGATGGCAATCG  GAGGACCAAAACGAACGAAATT |
| *Shp* | Forward  Reverse | GATCCTCTTCAACCCAGATGT  GCCATGAGGAGGATTCGGG |
| *Glp1* | Forward  Reverse | GGCACATTCACCAGCGACTAC  CAATGGCGACTTCTTCTGGG |
| *Slc10a2* | Forward  Reverse | GTCTGTCCCCCAAATGCAACT  CACCCCATAGAAAACATCACC |
| *Cyp8b1* | Forward  Reverse | CCAAGTGCCCCGGCAGGTTC  TCACTGCAGGGGCTTCAGGC |
| *Igfbp2* | Forward  Reverse | CAGACGCTACGCTGCTATCC  CCCTCAGAGTGGTCGTCATCA |
| *Tbp* | Forward  Reverse | CTTCCTGCCACAATGTCACAG  CCTTTCTCATGCTTGCTTCTCTG |
